# The correlation between radiative surface defect states and high color rendering index from ZnO nanotubes

**DOI:** 10.1186/1556-276X-6-513

**Published:** 2011-08-30

**Authors:** Jamil R Sadaf, Muhammad Q Israr, Omer Nur, Magnus Willander, Yong Ding, Zhong L Wang

**Affiliations:** 1 Department of Science and Technology, Campus Norrköping, Linköping University, SE-601 74 Norrköping, Sweden; 2 School of Materials Science and Engineering, Georgia Institute of Technology, Atlanta, GA 30332-0245 USA

**Keywords:** ZnO nanotubes, ZnO/GaN heterostructure, radiative surface defects, color rendering index, R9 color indexed

## Abstract

Combined surface, structural and opto-electrical investigations are drawn from the chemically fashioned ZnO nanotubes and its heterostructure with p-GaN film. A strong correlation has been found between the formation of radiative surface defect states in the nanotubes and the pure cool white light possessing averaged eight color rendering index value of 96 with appropriate color temperature. Highly important deep-red color index value has been realized > 95 which has the capability to render and reproduce natural and vivid colors accurately. Diverse types of deep defect states and their relative contribution to the corresponding wavelengths in the broad emission band is suggested.

## Introduction

The solid-state lighting holds tremendous prospective for future illumination, backlight panel display industry and biomedical applications due to their brightness and durability [1-3]. Over the past decade, much attention has been drawn towards white-light-emitting diodes (WLEDs) as new light sources due to their reliability with great economic and ecological consequences. So far, different materials and a number of nanostructures are being used to fabricate WLEDs such as phosphors, nanocrystals, polymers, and nanocrystal-polymer combination [4-7]. To this end, phosphor and polymers are being studied comprehensively for wavelength conversion and to generate full-color emission but still much efforts are required to achieve the light-emitting devices with high color rendering index (CRI) value approaching 100 for future lighting.

During the last years, zinc oxide (ZnO) material has been extensively investigated as a suitable contender for new-generation photonic devices. ZnO contains a promising emission tendency for blue/ultraviolet and full-color lighting, owing to the wide band gap, large exciton binding energy and many radiative deep levels depending on its synthesizing techniques [8,9]. The ease in the fabrication of nanoscale structures with huge diversity in shape and size is another advantageous characteristic of the ZnO material. However, the self-compensation feature of p-ZnO exists as a real hurdle in the pursuit of stable homojunctions of ZnO [10]. In this regard, GaN provides a suitable replacement of the p-ZnO for the fabrication of pn-heterostructures due to their better match in crystal structure, wide band gap and opto-electronic properties compared to other p-type materials. Among a variety of nanoscale structures of ZnO, nanotubes along with p-GaN have the potential to provide a heterostructure with substantial advantages and the conjunction of high surface to volume ratio with huge number of intrinsic and extrinsic defects could culminate a full-color illumination. Moreover, ZnO-nanotubes/GaN heterostructure have an aptitude to produce an environmentally benign alternative of the traditional lighting sources with high CRI value encompassing the diverse applications. Along with the first eight colors rendering indices of CRI (Ra), deep-red rendering index R9 contains a significant importance for the reproduction of the original colors of different objects. Furthermore, the heterostructure under investigation is based on simple manufacturing technique and offers high stability of the CRI with increasing temperature which is the main dilemma of the polymeric and phosphoric-based light-emitting devices. Here, a heterostructure fashioned with the combination of chemically fabricated ZnO nanotubes and Mg-doped GaN thin film has been used to unreveal the defect-related broad visible emission mechanism. Transmission electron microscope (TEM), cathodo- and electroluminescence (CL and EL) techniques have been utilized to observe the influence of the etching mechanism on the defect states in the nanotubes. Moreover, the corresponding impact of chemical etching on the radiative and non-radiative recombination has been studied which play a crucially important role in the production of high CRI and R9 values.

## Experimental

To make n-ZnO nanotubes/p-GaN heterostructure structure, vertically well-aligned ZnO nanorods have been grown on p-GaN thin film employing a low-temperature aqueous chemical synthesis technique. These nanorods have been further dipped in potassium chloride solution with concentration of 5 M for 10 h for the fabrication of nanotubes under the process of the wet chemical etching [11]. An insulating layer of Shipley 1805 (Shipley Company, Marlborough, MA, USA) has been spun coated to fill the space between the nanotubes for the isolation of electrical contacts followed by reactive ion etching to expose the tips of nanotubes. Finally ohmic contacts on p-GaN and n-ZnO have been made by thermal evaporation of the Ni/Au and Ti/Au bilayer electrodes, respectively.

## Results and discussion

**Figure 1:**
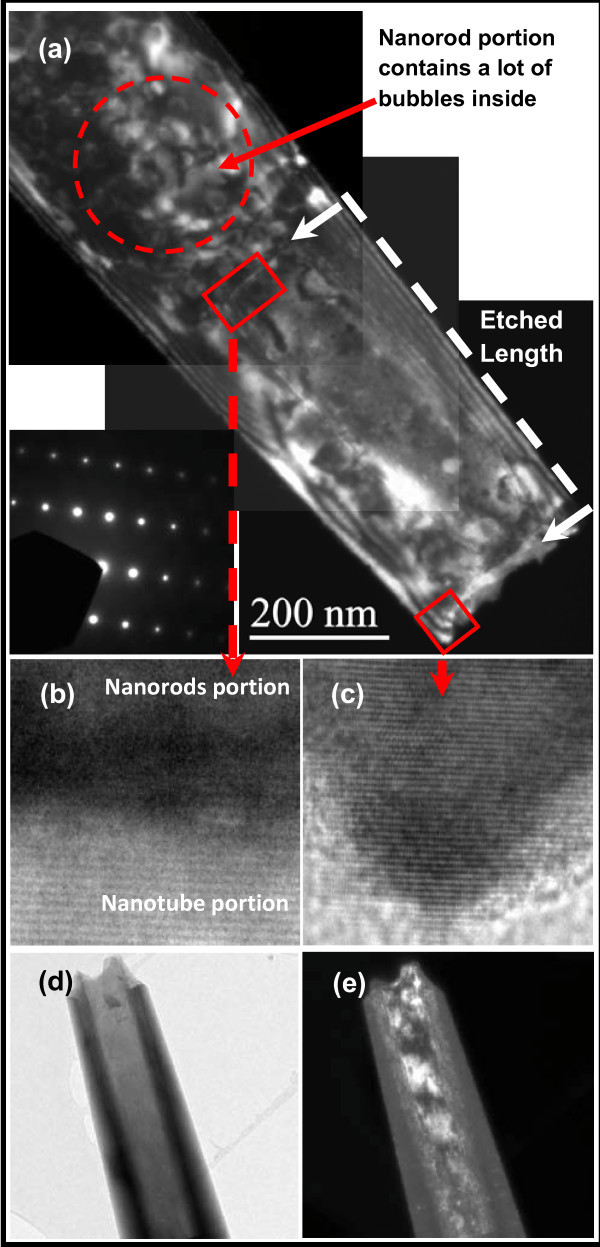
**TEM images of ZnO nanorods and nanotubes**. **(a) **LRTEM image of partially etched ZnO nanorod (white arrow). Non-etched part of the ZnO nanorod contains a lot of bubbles in its core (red dotted circle). The insert shows SAED pattern indicating the growth orientation along [0001]. **(b, c) **HRTEM images from different spots (red squares). **(d, e) **Comparative analysis of surface defects distribution on the walls of same nanotube from bright and dark field images.

Figure 1a depicts a low-resolution dark field TEM (LRTEM) image of half-way-etched single ZnO nanorod to observe the defect states concentrations in prior to and post-etched portions. It is observed that the core of the nanorod contains a lot of small bubbles; however, these bubbles disappear in the post-etched portion. It could be concluded that etching of nanorods is responsible for the elimination of defects states from the core of these nanorods. This is in accord with previously reported results about the presence of higher density of the defects in the central core of nanorods [12]. As the etching process is strongly concerned with the difference in stability between the polar and non-polar planes of ZnO nanorods, thus the preferential-etching of the meta-stable planes (polar planes) enables dissolution of the defect-rich central core of nanorod. Selected area electron diffraction (SAED) in the inset of Figure 1a illustrates that the crystal growth orientation of the nanotubes is along the [0001] which is preferred orientation for hexagonal ZnO structure. High-resolution TEM (HRTEM) images recorded from different spots in the nanotube depict good crystallinity of the nanostructure (Figure 1b, c). A smooth and clear bright-field image confirms impurity free nanowalls while a large number of intrinsic surface defect states are observed in the dark-field image which could be formed during the etching process (Figure 1d, e).

**Figure 2:**
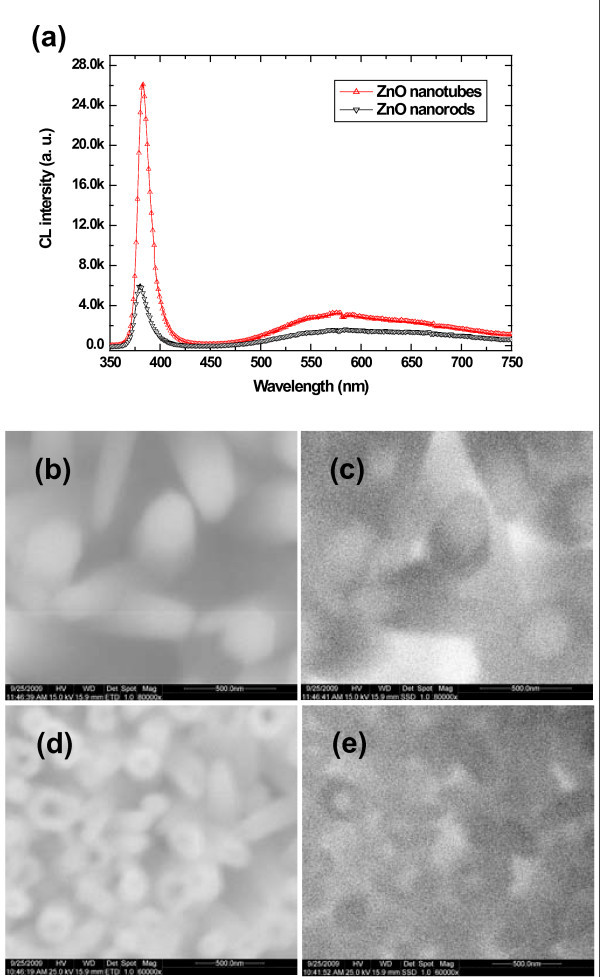
**CL spectra of ZnO nanorods and nanotubes**. **(a) **Room temperature CL spectra of ZnO nanorods (black) and nanotubes (red). **(b, d) **SEM images of the rods and tubes with their corresponding monochromatic CL images **(c, e)**.

Figure 2a shows a comparative analysis of the CL emission spectra recorded from ZnO nanorods and nanotubes. The main features of the spectra illustrate firstly the UV emission intensity, which is generally ascribed as originated from band edge of ZnO, from the nanotubes is much higher than the nanorods [13,14]. The reason of the strong emission of the UV could be assigned to the entrance of the electron beam into the nanotube where it can travel adopting a helical path by striking again and again with the inner surface of the nanotube. Secondly, the enhanced emission intensity in the visible range can be attributed to the higher concentration of surface defect states on the walls of the nanotubes [12,15]. Figure 2b, d shows the SEM images of solid nanorods and hollow nanotubes with their corresponding monochromatic CL images taken at a wavelength of 375 nm using an acceleration voltage of 10 KeV, Figure 2c, e. By combining the TEM and the CL results, we can conclude that the presence of small bubbles in the central core of the ZnO nanorods could be responsible for the non-radiative recombination which can suppress the visible emission. In the case of the nanotubes, the etching mechanism not only removes non-radiative recombination centers present in the central core but also generates the surface defect states on the walls of the nanotube along with the increase in surface area to volume ratio compared to nanorods. These originated surface defect states can act as additional radiative recombination centers and it is also a well-known fact that the presence of surface defect states is always higher in concentration compared to the core defect states [15].

**Figure 3:**
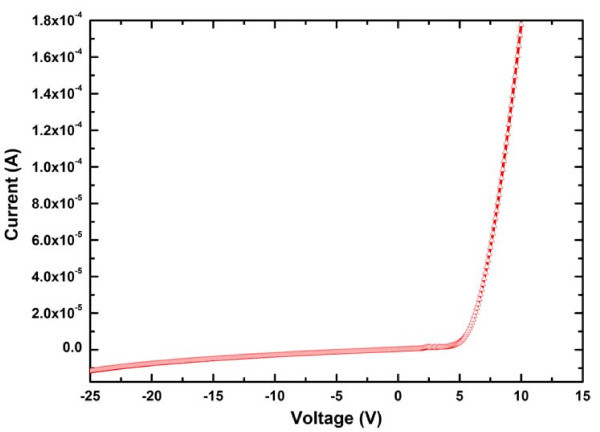
**I-V characteristic of ZnO nanotubes/GaN heterostructure**.

**Figure 4:**
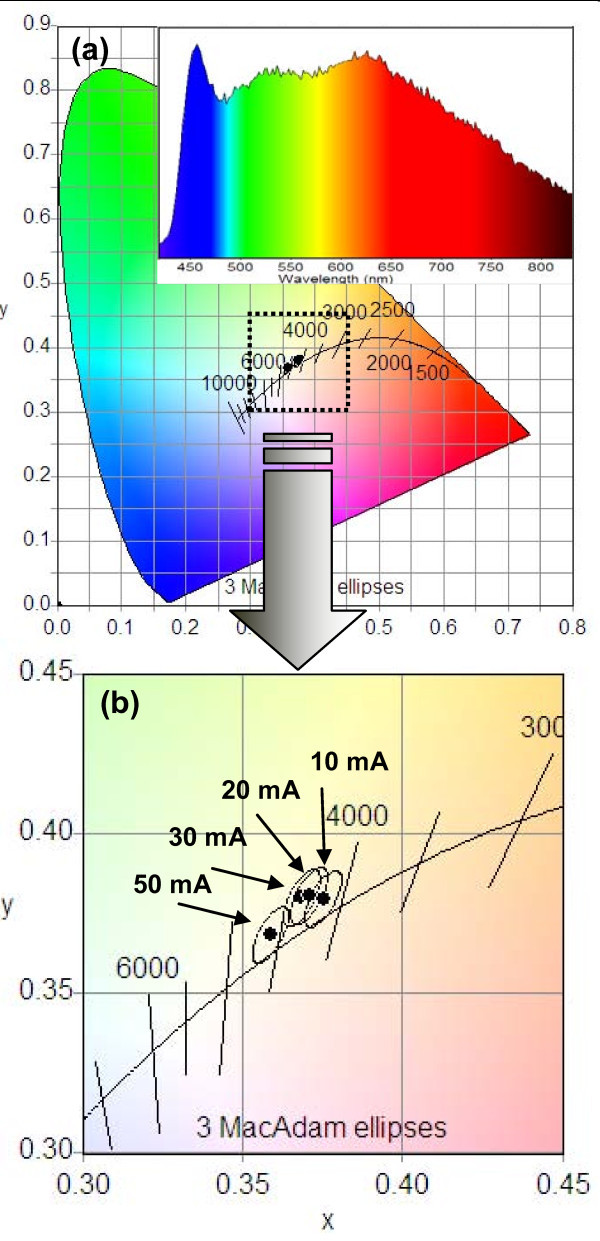
**CRI values corresponding to different injected currents**. **(a) **Chromaticity diagram shows high CRI values lying close to the Planckian locus. The insert shows the EL emission spectrum of the heterostructure LED. **(b) **High magnified image showing the CRI values at different operating currents (10, 20, 30, 50 mA).

The current-voltage (I-V) characteristics of the ZnO nanotubes/GaN film heterostructure LED reveal a good rectifying behavior, with a turn on voltage of approximately 5 V (Figure 3). The chromaticity diagram (CIE 1931) has been utilized to portray the color quality of the operating device which is generally considered good if the chromaticity coordinates lies near the Planckian locus (standard chromaticity coordinates of a blackbody). However, according to display applications, the quality of the visible emission depends not only on the position of the CRI in the chromaticity diagram but appropriate color temperature is also an important factor. The chromaticity diagram of the presented device depicts that the emission coordinates are very close to the locus indicating that the LED is emitting almost perfect white light with a CRI value of 96 which is a result of high fidelity and good rendering of different colors. In addition, a color temperature in the range of 4,100 to 4,600 K is also coherent to the sunlight for the cool light, Figure 4a. These CRI values have been extracted from the room temperature EL spectrum which depicts three clear emission peaks covering the whole visible region from 400 to 830 nm, insert of Figure 4a. This broad emission band from the ZnO nanotubes/GaN film heterostructure LED is generally related to the fabrication process of nanotubes with a low temperature regime which produces a large number of defects with high diversity. The emission peak at around 450 nm is being originated from the electron-hole recombination at the ZnO/GaN interface of the LED [16]. The green emission peak, centered at around 530 nm, could be ascribed to the presence of intrinsic defect states such as singly ionized oxygen vacancies. The depleted region on the surface of ZnO along with these oxygen vacancies must be responsible for the green emission due to plausible recombination process when the device is biased [17]. Additionally, the inner and outer surfaces of the hollow nanotubes possess a higher density of oxygen vacancies due to the high porosity compared to solid nanorods [18]. The orange-red emission peak can partially be attributed to the presence of extrinsic defects in the nanotubes and heavily Mg-doped GaN film as well as intrinsic defects in the nanotubes produced during the etching process [19,11]. However, the contribution from the GaN in orange-red peak could come through the transition between the deep acceptors and deep donors. In addition, one could expect the activation of the defect states discussed above by the UV emission and the re-absorbance in the ZnO. The defect-related emission can be further enhanced by the recombination process in the nanotubes, when the device is biased. Table 1 summarizes the coordinates and color rendering indices of the ZnO nanotubes/GaN heterostructure along with their corresponding correlated color temperatures. Obviously, the CRI values demonstrate good stability under different values of injection current in the range from 10 to 50 mA producing cool light in the color temperature ranging from 4,100 to 4,600 K shown in the magnified chromaticity diagram, Figure 4b. One of the most important aspects of the presented LED is very high values (95 to 98) of special rendering index R9 with deep-red saturated color which enhances the skills of device precisely for the reproduction of natural and vivid colors.

**Table 1:** Color rendering index, color temperature, R9 and x, y coordinates values corresponding to different injection currents

Injected current (mA)	Color temperature	Color rendering index	R9	*X*-coordinate	*Y*-coordinate
50	4,586	96	98	0.3589	0.3683
30	4,355	96	96	0.3686	0.3798
20	4,291	96	95	0.3709	0.3804
10	4,162	97	98	0.3753	0.3795

## Conclusion

In summary, we have correlated the removal of non-radiative recombination centers present in the core of nanorods as well as the production of surface defect states as radiative recombination centers in nanotubes and their role in the enhancement in the emission intensity and CRI value of the heterostructure. The broad band emission spectrum is suggested as a result of the superposition of different emission peaks corresponding to the diversity of the deep level defect states. A high value of R9 > 95 has been achieved which could uncover the device applications in the fields of decorative industry and medical surgery.

## Competing interests

The authors declare that they have no competing interests.

## Authors' contributions

JRS, MQI, ON and MW initiated the presented study, provided current-voltage curve, cathodo- and electroluminescence measurements, calculated the color rendering indices of the light emitting device and wrote the manuscript. YD and ZLW provided all the measured results from transmission electron microscope. All the authors participated in the revision and approval of the manuscript.
